# Exploring the Tryptophan Metabolic Pathways in Migraine-Related Mechanisms

**DOI:** 10.3390/cells11233795

**Published:** 2022-11-27

**Authors:** Tamás Körtési, Eleonóra Spekker, László Vécsei

**Affiliations:** 1 Faculty of Health Sciences and Social Studies, University of Szeged, Temesvári krt. 31, H-6726 Szeged, Hungary; 2 ELKH-SZTE Neuroscience Research Group, University of Szeged, H-6725 Szeged, Hungary; 3 Department of Neurology, Faculty of Medicine, Albert Szent-Györgyi Clinical Center, University of Szeged, H-6725 Szeged, Hungary

**Keywords:** primary headaches, migraine, tryptophan, serotonin pathway, kynurenic pathway, serotonin, melatonin, kynurenic acid, PACAP, CGRP

## Abstract

Migraine is a complex neurovascular disorder, which causes intense socioeconomic problems worldwide. The pathophysiology of disease is enigmatic; accordingly, therapy is not sufficient. In recent years, migraine research focused on tryptophan, which is metabolized via two main pathways, the serotonin and kynurenine pathways, both of which produce neuroactive molecules that influence pain processing and stress response by disturbing neural and brain hypersensitivity and by interacting with molecules that control vascular and inflammatory actions. Serotonin has a role in trigeminal pain processing, and melatonin, which is another product of this pathway, also has a role in these processes. One of the end products of the kynurenine pathway is kynurenic acid (KYNA), which can decrease the overexpression of migraine-related neuropeptides in experimental conditions. However, the ability of KYNA to cross the blood–brain barrier is minimal, necessitating the development of synthetic analogs with potentially better pharmacokinetic properties to exploit its therapeutic potential. This review summarizes the main translational and clinical findings on tryptophan metabolism and certain neuropeptides, as well as therapeutic options that may be useful in the prevention and treatment of migraine.

## 1. Migraine

Migraine is one of the most common neurological conditions with a high prevalence and morbidity [1] and is associated with a high economic burden [2]. The estimation of the Migraine Impact Model projected approximately 60,000–686,000 annual workdays as being affected by lost productive time due to migraine and estimated annual indirect costs as totaling 6.2–8.5 times the annual direct costs in USA [3]. Clinically, migraine is characterized by a unilateral throbbing, pulsing headache, associated with various symptoms, such as allodynia, photophobia, and phonophobia, which lasts for hours to days, and the pain has a negative impact on daily activities [4].

Despite extensive research, there are still questions that have not been fully answered about the pathomechanism of migraine; however, translational and clinical trials suggest that activation and sensitization of the trigeminal system (TS) are important during the attacks [5]. The theory of TS constitutes neurovascular incidence, peripheral and central sensitization, and neurogenic inflammation in the dural vessels. According to the literature, the major contributing pathophysiological event thought to initiate migraine is cerebral and meningeal arterial vasodilation. Nevertheless, the role of vasodilation in migraine is not fully understood, and recent findings challenge its necessity. During the attacks, several mediators are released from blood vessels, such as growth factors, cytokines, adenosine triphosphate (ATP), and nitric oxide (NO), which induce local sterile meningeal inflammation [6,7].

Glutamate is an important excitatory neurotransmitter in the central nervous system (CNS), and it plays a role in pain transmission, central sensitization, and cortical spreading depolarization [8,9,10]. Increased glutamate levels have been noticed in blood and cerebrospinal fluid both interictally and ictally in migraine patients [11,12,13]; thus, they are involved in migraine pathophysiology.

In addition to glutamate, other neurotransmitters are involved in the development of attacks. Serotonin (5-HT) has a vasoconstrictor effect on blood vessels, thereby affecting nociceptive pain [14]. 5-HT receptors are present in the TS and cranial vessels [15,16], and their agonists, i.e., triptans, are effective for migraine relief [17,18]. Accordingly, neurotransmission mediated by 5-HT is also involved in migraine [19]. 

**Figure 1:**
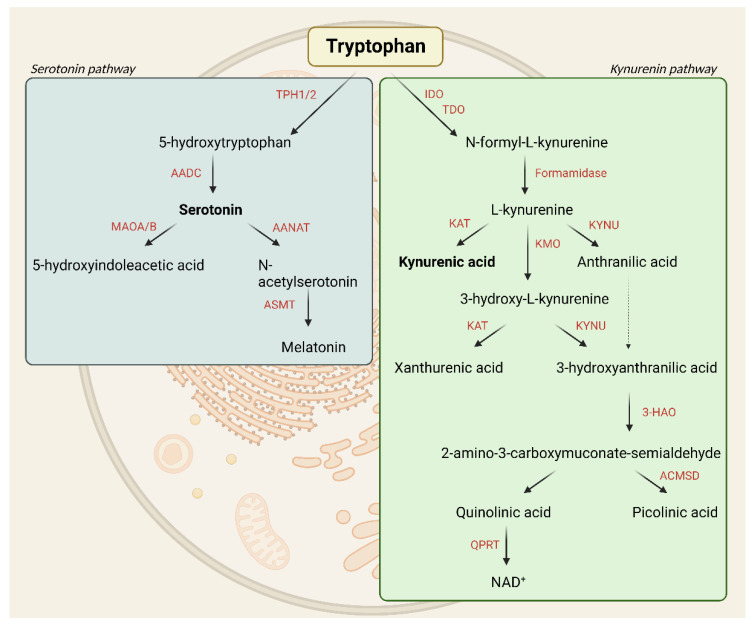
The two main pathways of tryptophan metabolism: serotonin and kynurenine pathways.

Tryptophan is an essential amino acid required for different metabolic reactions among others, such as 5-HT production [20]; however, only a small amount of 5-HT is formed during tryptophan metabolism. The kynurenine pathway (KP) is responsible for 95% of tryptophan metabolism, which is closely related to both glutamatergic and serotonergic mechanisms; thus, the catabolites of this pathway are the focus of migraine research (Figure 1).

This review article summarizes the emerging evidence supporting the involvement of tryptophan metabolism in the pathophysiology of migraine, as well as presents the latest results of preclinical research and the therapeutic possibilities of the disease.

## 2. Tryptophan and Its Role in Migraine

Tryptophan is an essential amino acid needed to produce and maintain proteins, muscles, enzymes, and neurotransmitters. Changes in tryptophan levels can cause an imbalance in the synthesis of 5-HT and melatonin in the brain and may play a role in the pathophysiology of numerous neuropsychiatric and neurodegenerative disorders [21].

Some research groups observed decreased serum and plasma tryptophan levels in migraine sufferers compared to healthy controls [22,23]. Furthermore, other clinical investigations showed increased tryptophan levels in migraine, especially during the aura phase [24,25]. Similarly, increased serum tryptophan was reported in cluster headaches [26]. 

Several studies have confirmed a reduction in tryptophan level in the interictal period and an increase in the ictal phase of migraine patients [27,28]. Tryptophan depletion does not trigger migraine attacks but causes lower levels of 5-HT in the brain, which enhance symptoms of migraine [29,30,31]. In a study, tryptophan depletion induced headache in migraineurs and increased nausea and dizziness. Moreover, ratings of glare and light-induced pain were greater in the tryptophan depletion condition [32]. Consistent with the results above, Jahromi et al. demonstrated that increased tryptophan intake reduces migraine attacks [33,34]. 

The fact that tryptophan is the precursor of several components that are possibly involved in migraine pathogenesis (e.g., 5-HT and kynurenines) can explain the relationship between tryptophan and migraine (Figure 1).

## 3. Role of the Tryptophan/Serotonin Pathway in Migraine

5-HT was first identified as a vasoconstrictor present in the blood [35], which constricts blood vessels, thereby potentially modulating nociceptive pain [14,36]. 5-HT receptors can be classified into seven families, which can be further divided into 14 subtypes, all of which are members of the G-protein-coupled receptor family, except the 5-HT_3_ receptor, which is a ligand-gated ion channel [37]. 5-HT receptors are widely distributed in the CNS, including several areas involved in migraine, such as the striatum, cortex, hippocampus, thalamus, cerebellum, and raphe nuclei [38,39].

Sicuteri et al. were the first to suggest the importance of 5-HT in migraine when they found that, during a migraine attack, the amount of 5-hydroxyindoleacetic acid (5-HIAA), considered the main metabolite of 5-HT, increased in the urine, while the platelet 5-HT concentration decreased [40,41]; these results were confirmed by Curran et al. [42]. Other studies have reported that 5-HT infusion can interrupt spontaneous [43] or reserpine-induced [44] headache. Ren et al. reported low levels of serum serotonin in migraine patients, which was consistent with previous studies [45,46]. Moreover, they also found low levels of tryptophan in these patients [22].

### 3.1. Serotonin Pathway

**Figure 2:**
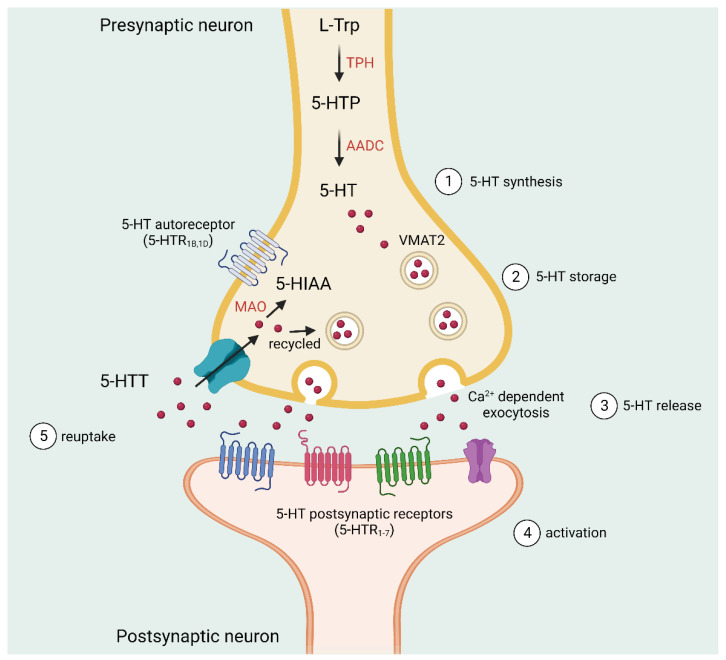
Serotonin pathway. (1) L-tryptophan is converted to 5-HT by TPH and AADC enzymes. (2) 5-HT is then taken up into vesicles in the axon terminal via VMAT2. (3) After an action potential, 5-HT is released into the synapse. 5-HT can also interact with presynaptic and postsynaptic receptors. (4) All 5-HT receptors are post-synaptically expressed on non-serotonergic neurons, and autoreceptors are located pre-synaptically on the serotonergic neurons. (5) Free 5-HT is removed from the synapse by 5-HTT, which controls the extent and duration of 5-HT receptor activation. Furthermore, 5-HT can be metabolized by MAO and aldehyde dehydrogenase into 5-HIAA, which is excreted in the urine. L-Trp: L-tryptophan, TPH: L-tryptophan hydroxylase, AADC: L-aromatic amino acid decarboxylase, 5-HTP: 5-hydroxytryptophan, 5-HT: serotonin, VMAT2: vesicular monoamine transporter isoform 2, 5-HTT: serotonin transporter, 5-HIAA: 5-hydroxyindoleacetic acid, MAO: monoamine oxidase.

The biochemical pathway for 5-HT synthesis initially involves the transformation of L-tryptophan into 5-hydroxytryptophan (5-HTP) by the rate-limiting enzyme L-tryptophan hydroxylase (TPH). 5-HTP is then decarboxylated to become 5-HT via the action of the cytosolic enzyme L-aromatic amino acid decarboxylase (AADC) [47,48]. Extracellular 5-HT enters the cells using the serotonin transporter (5-HTT), and excess 5-HT is metabolized. The metabolism of serotonin is primarily carried out by the outer mitochondrial membrane enzyme, monoamine oxidase (MAO) [49,50]. Finally, with the help of an aldehyde dehydrogenase enzyme, it is converted into 5-HIAA, which is excreted in the urine [47] (Figure 2).

### 3.2. Serotonin Transporter

5-HTT retakes 5-HT from the synaptic gap to the presynaptic terminals, thereby reducing the effect of 5-HT. The transport process is controlled by the Na+/Cl^−^ ion gradient [51]. 5-HTT occurs mainly in the area of the raphe nuclei and serotonergic projection areas (e.g., cortical areas, thalamus, hippocampus CA3 region, and amygdala) [52]. Imaging studies have established that the distribution of 5-HTT in the brain stem area is greater in migraine patients [53]. It has been observed that familial hemiplegic migraine (FHM) patients have a low level of 5-HT in platelets, and it has also been described that the 5-HT transport capacity is low. In addition, reduced metabolite levels in cerebrospinal fluid were observed in these patients [54]. 

### 3.3. Serotonin Receptors

5-HT receptors are important in the regulation of serotonergic neurotransmission, and they play a distinguished role in several behavioral and physiological functions [55]. In previous studies, it was observed that the neurons of the dorsal raphe and the trigeminal ganglia (TG) are mostly serotonergic [16,56]. 

In humans, it has been demonstrated that both receptor 5HT_1B_ and 5HT_1D_ subtypes are present in trigeminal neurons [57,58], and both receptors have been detected at mRNA and protein levels in the TG [59] and colocalize with calcitonin gene-related peptide (CGRP), substance P (SP), and nitric oxide synthases (NOS) [58]. 

Triptans are 5-HT_1B/1D_ agonists with some affinity for the 5-HT_1F_ receptor subtype, and they are clinically effective anti-migraine drugs. They can have an inhibitory effect on the trigeminal sensory fibers, which is attributed to the inhibition of endogenous CGRP and SP release [60]. The efficacy of triptans also suggests that 5-HT may modulate the pathogenesis of migraine. Unfortunately, triptans are contraindicated in patients with high blood pressure and cardiovascular or cerebrovascular disease due to their vasoconstrictive effect. In addition, these drugs are not effective for everyone, often leading to excessive drug use, which eventually causes migraines to become chronic [61].

These facts led to the development of ditans, the new class of selective 5-HT_1F_ receptor agonists that do not have vasoconstrictive properties [62,63]. The 5-HT_1F_ receptor is expressed in several brain areas involved in migraine attacks, such as the cortex, the hypothalamus, the trigeminal ganglia, the trigeminal nucleus caudalis (TNC), the locus coeruleus, the middle cerebral artery, and the upper cervical cord [64,65]. Several selective 5-HT_1F_ receptor agonists have been developed in the past years; in preclinical studies, they could successfully inhibit dural extravasation after TG stimulation and hinder neuronal activation in the TNC following trigeminovascular activation [66,67,68,69]. However, only lasmiditan can currently be used as anti-migraine therapy, but it has no therapeutic gain over triptans. Lasmiditan can cross the BBB and, thus, exert its effects centrally on the trigeminovascular system; however, at the same time, it also has a peripheral effect, via 5-HT_1F_ receptors expressed on trigeminal afferents or TG [70]. Lasmiditan can probably moderate the activation of Sp5C second-order trigeminal neurons, which has an important role in the pathomechanism of migraine [71,72].

5-HT_2B_ receptors can influence the release of 5-HT through the 5-HTT and are also involved in the normal physiological regulation of blood plasma 5-HT levels [73]. In rats, 5-HT_2B_ receptors are slightly expressed in neurons located in the cerebellum, the posterior hypothalamus, the lateral septum, the medial amygdala, the spinal cord, and the dorsal root ganglion (DRG). Unlike the 5-HT_1_ receptor, it seems that the 5-HT_2B_ receptors do not inhibit/decrease the release of neuropeptides involved in migraine (CGRP, glutamate) from trigeminal neurons [74]. Indeed, the 5-HT_2B_ receptor can activate NOS, which promotes the synthesis of NO [75], a potentially key component in the development of a migraine attack. In guinea pigs, acute activation of 5-HT_2B_ receptors by m-chlorophenylpiperazine (mCPP) led to NO-dependent plasma protein extravasation (PPE) in the dura mater and neuronal activation in the TNC, which could be inhibited by selective 5-HT_2B_ receptor antagonists [76,77,78]. In humans, mCPP, with 5-HT_2B/2C_ receptor affinity, leads to delayed migraine-like headaches in migraine sufferers and nonspecific headaches in healthy subjects [79]. Methysergide, a 5-HT_2B_ antagonist, can reduce the frequency of migraine, but it has to be used for a longer period to exert its therapeutic effect [80]. Johnson et al. reported that, after electrical stimulation of the TG, LY202146, a selective 5-HT_2B_ receptor antagonist, failed to inhibit protein extravasation [77], suggesting that the 5-HT_2B_ receptor may play a role in triggering the migraine attack, but is not related directly to the release of peptides from trigeminal neurons. These observations resemble the results obtained in clinical research where effective preventive agents, such as methysergide and pizotifen, could not inhibit the onset of a migraine attack.

**Figure 3:**
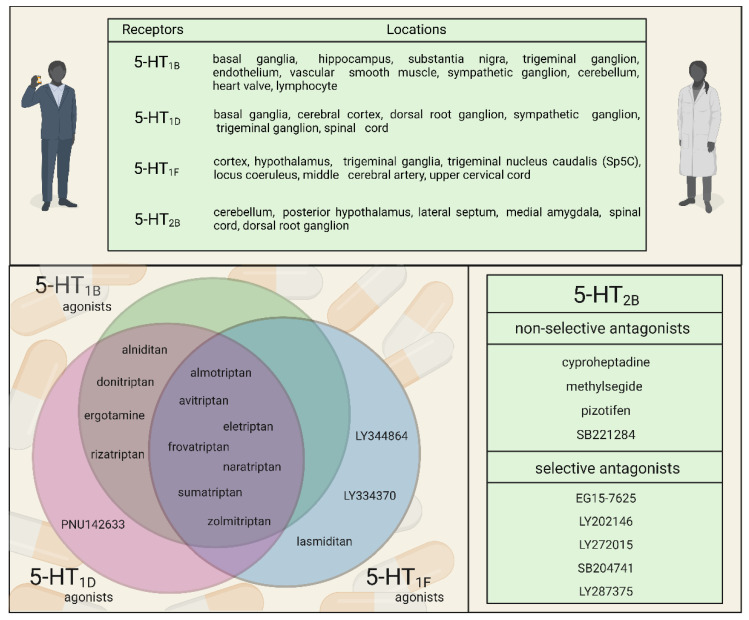
5-HT receptors and their relevance in migraine therapy.

On this basis, it was suggested that meningeal 5-HT_2B_ receptors may play a role in the onset of migraine attacks (Figure 3). 

### 3.4. Melatonin

Melatonin is a tryptophan metabolite that plays a role in regulating circadian rhythms, and numerous studies have demonstrated that melatonin can exert its anti-migraine effect in several ways. Melatonin can regulate neurotransmitters and neural pathways; it can inhibit the synthesis of NO, as well as the release of CGRP and dopamine, and it can antagonize glutamate-induced excitotoxicity [81,82,83,84]. Furthermore, it has an anti-free radical effect and inhibits the release of inflammatory factors [85]. It is supported by many studies that melatonin has a role in pain transmission and sensitization [84,86,87,88,89]. Membrane melatonin receptors (MT1 and MT2) have been identified in the thalamus, dorsal horn of the spinal cord, trigeminal tract, and trigeminal nucleus, which are involved in nociceptive transmission [90,91]. 

**Figure 4:**
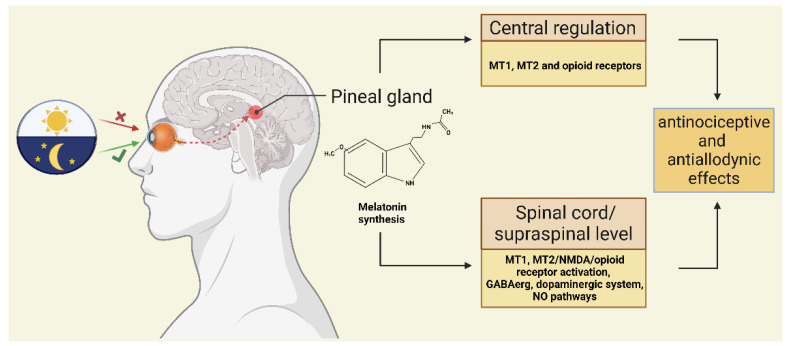
Melatonin synthesis and its anti-nociceptive and anti-allodynic effects. Melatonin can probably induce an anti-nociceptive effect through the regulation of MT1/MT2 receptors in the spinal cord and brain. It also interacts with other receptors such as NMDA, opioids, the dopaminergic system, the GABAergic system, and the NO pathway to exert anti-nociceptive and anti-allodynic effects. MT1/2: melatonin receptor 1/2, NMDA: N-methyl-D-aspartate, GABA: gamma-aminobutyric acid, NO: nitric oxide.

Melatonin can increase the release of β-endorphin from the pituitary gland and interacts with opioidergic, muscarinic, nicotinic, serotonergic, and α1 and α2-adrenergic receptors located in the CNS and the dorsal horn of the spinal cord; thus, it may be able to exert an analgesic effect [92,93,94] (Figure 4). In fibromyalgia, inflammatory bowel syndrome, and migraine, melatonin was able to reduce pain [95,96,97]. In another study, melatonin treatment was able to modify the central level of brain-derived neurotrophic factor (BDNF) in rats submitted to acute and chronic inflammation [98].

Masruha and colleagues found low levels of 6-sulfatoxymelatonin—a urinary metabolite of melatonin—in migraine patients [99]. Previous studies found low levels of melatonin in episodic [100] and chronic [101] migraine patients. Murialdo et al. found that, during the luteal phase, migraineurs showed a less pronounced change in melatonin levels than controls. Melatonin secretion was further decreased during migraine attack [102]. In line with these observations, Brun et al. found significantly lower melatonin levels in women with migraine during the cycle, while healthy participants showed a significant increase in melatonin secretion from the follicular to the luteal phase [103].

According to these data, it is possible that melatonin may be beneficial in migraine prophylaxis.

## 4. Role of Tryptophan/Kynurenine Pathway in Migraine

The role of the tryptophan/kynurenine metabolic pathway is receiving more attention in various illnesses including migraine [48]. In parallel to 5-HT synthesis, the central route of the tryptophan metabolism is the KP [104].

### 4.1. Kynurenine Pathway

**Figure 5:**
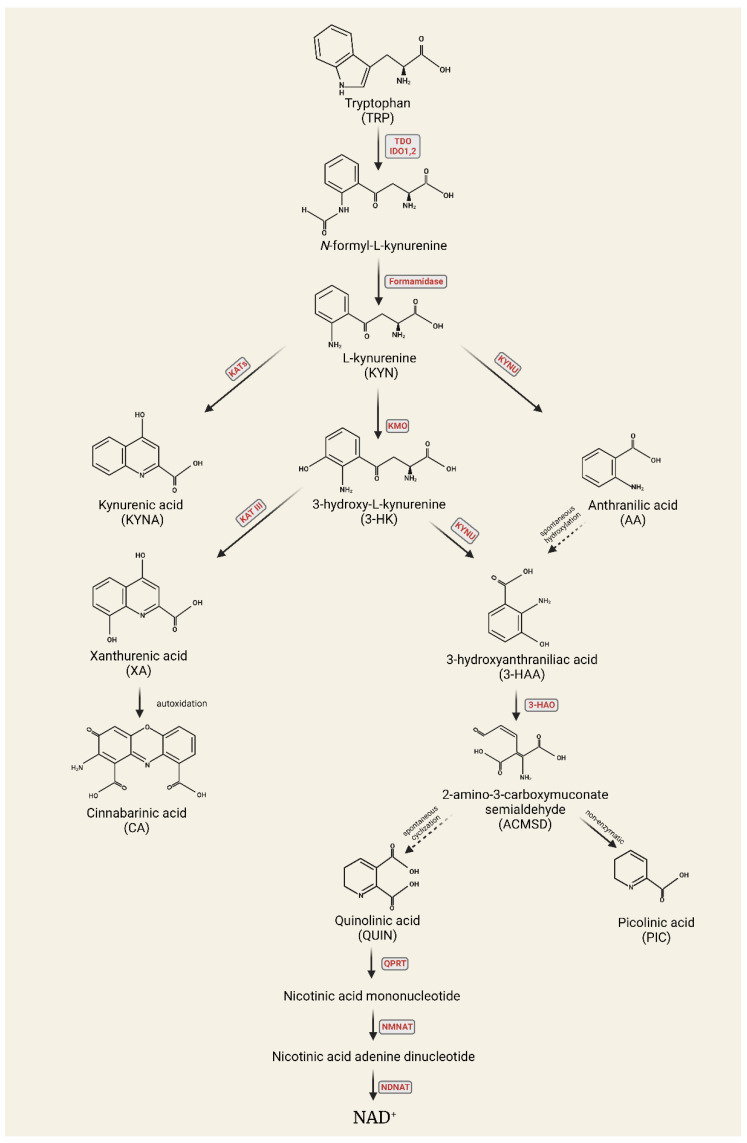
Kynurenine pathway.

The transformation process of tryptophan into N-formyl-L-kynurenine is carried out by two rate-limiting enzymes: tryptophan-2,3-dioxygenase (TDO) and indoleamine-2,3-dioxygenase (IDO). N-formyl-L-kynurenine is degraded by formamidase to L-kynurenine (L-KYN). L-KYN can be metabolized into kynurenic acid (KYNA), 3-hydroxy-L-kynurenine (3-HK), or anthranilic acid (AA) under the action of kynurenine aminotransferase (KAT), kynurenine-3-monooxygenase (KMO), and kynureninase (KYNU) enzymes. 3-HK can be further converted to xanthurenic acid (XA) by KAT or to 3-hydroxyanthranilic acid (3-HANA) by KYNU. 3-Hydroxyanthranilic acid is then metabolized by 3-hydroxyanthranilate oxidase (3-HAO) to 2-amino-3-carboxymuconate-semialdehyde, which is transformed into picolinic acid (PIC) or quinolinic acid (QUIN). In the last step of the KP, QUIN is converted into the coenzymes nicotinamide adenine dinucleotide (NAD) and nicotinamide adenine dinucleotide phosphate (NADP) [105] (Figure 5).

### 4.2. Kynurenines

KP produces neuroactive metabolites which have a role in the modification of the trigemino-vascular activation processes and can interact with glutamate receptors in the CNS [106]; therefore, they may be involved in the pathophysiology of migraine.

Among the kynurenines, KYNA should be mentioned, which can act through N-methyl-D-aspartate (NMDA), α-amino-3-hydroxy-5-methyl-4-isoxazole propionic acid (AMPA), kainate receptors, and G-protein-coupled receptor 35 (GPR35), and these receptors have a major role in pain processing and neuroinflammation [105]. Experimental data suggest, that in the brain, an increased level of KYNA has neuroprotective effects [107,108]. Additionally, in an animal model of migraine, KYNA was able to inhibit trigemino-vascular activation [109,110]. Furthermore, KYNA can modulate the activation of migraine generators and inhibits cortical spreading depression (CSD) [19]. Oláh and colleagues reported that, in rats, peripherally administered KYNA was able to reduce the number of CSD waves; moreover, it decreased the permeability of the blood–brain barrier (BBB) during CSD [111]. Knyihár-Csillik et al. reported reduced KAT expression after the electrical stimulation of the TG [112]. Moreover, Spekker et al. found that inflammatory soup was able to cause sterile neurogenic inflammation in the dura mater and increased the area covered by CGRP and transient receptor potential vanilloid 1 (TRPV1) immunoreactive fibers, as well as the number of neuronal nNOS-positive cells in the caudal trigeminal nucleus, and pretreatment with KYNA was able to modulate the changes caused by inflammatory soup. KYNA probably inhibited the glutamate system, thereby preventing the sensitization processes which are key actors in migraine [113]. 

It has been reported that KYNA has anti-nociceptive effects in both the first- and second-order trigeminal nociceptors. Zhang et al. found that KYNA dose-dependently suppressed carrageenan-induced thermal hyperalgesia and significantly reduced c-fos expression in both the superficial and the deep laminae of the dorsal horn in rats [114]. In another study, after carrageenan injection into the tibio-tarsal joint, locally administered KYNA was able to abolish allodynia and cause anti-nociception [115]. 

The therapeutic use of KYNA is hampered by the fact that it is difficult to cross the BBB [116]. The development of KYNA analogs with retained or modified activity can be a solution to this problem. These compounds are promising because they are capable of selectively inhibiting NMDA receptors containing the NR2B subunit, which play a role in the modulation of pain perception.

L-KYN is the source of all the other kynurenine metabolites, and it is readily transported across the BBB [116]. L-KYN in combination with probenecid can prevent nitroglycerin (NTG)-induced changes in c-fos expression in rat TNC [109]. Peripheral treatment with L-KYN can dose-dependently enhance the concentration of KYNA in the brain; thus, it may provide a possible therapeutic solution for the treatment of several neurological disorders, including primary headaches. However, the physiological effect and safety of L-KYN in vivo in humans are still awaiting clarification.

## 5. Neuropeptides in Migraine

### 5.1. Pituitary Adenylate Cyclase-Activating Polypeptide 

Some neuropeptides play a role in neurogenic inflammation, thereby activating TS. The pituitary adenylate cyclase-activating polypeptide (PACAP) is a member of the vasoactive intestinal peptide (VIP)/secretin/glucagon peptide family [117]. PACAP is widely expressed in the human body, with extensive effects [118]. Literature data prove that this peptide plays roles such as neuromodulation [119] and neuroprotection [120], in addition to antiapoptotic effects [121] and differentiation-inducing effects in the developing nervous system [122]. The peptide has two biologically active forms; PACAP1-38, which consists of 38 amino acids, and PACAP1-27, which contains 27 amino acids at its N-terminus. These are produced by alternative splicing from the PACAP precursor, preproPACAP [123,124]. The effects of PACAP are mediated through three G-protein-coupled receptors: VPAC1, VPAC2, and PAC1. The latter is a high-affinity and PACAP-selective receptor, while VPAC1 and VPAC2 receptors show a comparable affinity to PACAP and VIP [125]. The PAC1 receptor has been shown to play crucial roles in the functioning of the nervous system. The activation of this receptor induces numerous signal transduction cascades, including phospholipase C, adenylyl cyclase, MEK/extracellular signal-regulated kinase (ERK), and Akt pathways that regulate a number of physiological systems to maintain functional homeostasis [126,127]. Previous studies evidence that, through PACAP activation, PAC1 receptor-mediated pathways are implicated in a number of disorders including depression, posttraumatic stress disorder, metabolic abnormalities, chronic pain, and migraine [128].

In recent years, several clinical investigations have reflected the possible relevance of PACAP in migraine. In experimental conditions, intraperitoneal administration of PACAP1-38 evoked notable photophobia and meningeal vasodilatation, as well as increased the number of c-fos-positive activated neurons in the brainstem in wildtype, but not in PACAP1-38-deficient mice [129]. Elevation of PACAP1-38 concentration was also detected in the brainstem after the activation of the TS in different animal models. The intraperitoneal administration of NTG also provoked an increase in PACAP1-38 and PACAP1-27 expression 3 h after the treatment in the TNC [130]. Furthermore, electrical stimulation of trigeminal ganglion (ES-TG) resulted in significantly increased PACAP1-38 immunoreactivity 3 h after ES-TG of the plasma and PACAP1-38 and PACAP1-27 immunoreactivity in the TNC [130]. The endogenous antagonists of NMDA receptor, KYNA and its synthetic analog SZR-72, were able to inhibit overexpression of PACAP at both the proteome and transcriptome levels, suggesting that KYNA and SZR-72 is a potential new drug candidate for PACAP-targeted headache therapy in the future. 

In patients suffering from migraines, the level of PACAP1-38 in the blood is increased during the migraine attack compared to the interictal period, suggesting a potential biomarker function of peptide in the disease [131]. Furthermore, intravenous administration of PACAP1-38 provoked headache and vasodilatation, in both healthy participants and migraine sufferers, whereas it delayed migraine-like attacks only in migraineurs [132,133]. In migraineurs without aura, the development of PACAP1-38-induced migraine-like attack was independent of the severity of the family load [134]. In the same study, 90 min after the PACAP treatment, the levels of numerous markers relevant to the disease (such as VIP, prolactin, S100B, and thyroid-stimulating hormone (TSH)) were increased in the plasma [134]. Correlation was shown between the microstructural integrity of the white matter and the interictal plasma PACAP1-38 immunoreactivity in migraineurs [135]. In addition, magnetic resonance imaging angiography examinations revealed that PACAP1-38 evoked headache was associated with prolonged vasodilatation of the middle meningeal artery (MMA), but not the middle cerebral artery (MCA) [136]. The anti-migraine drug sumatriptan was able to alleviate the headache, which mirrored the contraction of the MMA, but not the MCA, suggesting that PACAP1-38-induced headaches may arise from the extracerebral arteries.

### 5.2. Calcitonin Gene-Related Peptide

The “old warrior” CGRP is another pathogenic factor in the pathomechanism of migraine. A previous study confirmed that the expression of CGRP and SP was elevated during ES-TG of the external jugular vein of cats [137]. In addition to PACAP, CGRP can activate mast cells, leading to the secretion of vasoactive, proinflammatory, and neuro-sensitizing mediators, thereby contributing to the activation of TS [138,139] PACAP1-38 administration can cause increased CGRP expression in the brainstem, suggesting a possible link between CGRP and PACAP1-38 release [140]. CGRP and PACAP show co-expression; 23% of the neurons expressed both CGRP and PACAP1-38 in rat TRG, and CGRP (49%) was expressed in more neurons compared to PACAP1-38 (29%) [141]. In an experimental model of migraine, the simultaneous release of these neuropeptides was detected; a chronic NTG injection caused elevated concentrations of CGRP and PACAP in the plasma of rats, while the intervention resulted in mechanical and thermal hyperalgesia [142]. These data are consistent with our experimental results; orofacial complete Freund’s adjuvant (CFA) treatment caused significant CGRP and PACAP release in the brainstem. This elevation showed correlation with the mechanical hyperalgesia of animals [143]. However, activation of the TS is possible with different CFA treatments, which eventuates pain-associated pathological states, including migraine, neuralgias, and temporomandibular joint (TMJ) disorders [144]. A recent study examined the effect of CFA on the mitogen-activated protein kinase (MAPK) expression, which has a major role in the pain-related process. Administration of CFA in the TMJ resulted in significant ERK1/2 and p38 MAPK elevation in the TG [145]. Dural administration of CFA increased the expression of ERK1/2, interleukin-1 (IL-1β), and CGRP in the TG. In addition, high glutamate and c-fos immunoreactivity was observed in TNC and cervical neurons [146]. Following orofacial CFA treatment in the TG and TNC, CGRP, ionized calcium-binding adapter molecule 1 (Iba1) and glial fibrillary acidic protein (GFAP) gene expression changes were revealed, reflecting that CFA-induced neuroinflammation induces elevated CGRP and PACAP1-38 levels. [147]. Despite the similarities between CGRP and PACAP, experimental investigations suggest that these neuropeptides act independently, increasing their future therapeutic potential [148,149].

### 5.3. The Relationship between Neuropeptides and the Kynurenine System

**Figure 6:**
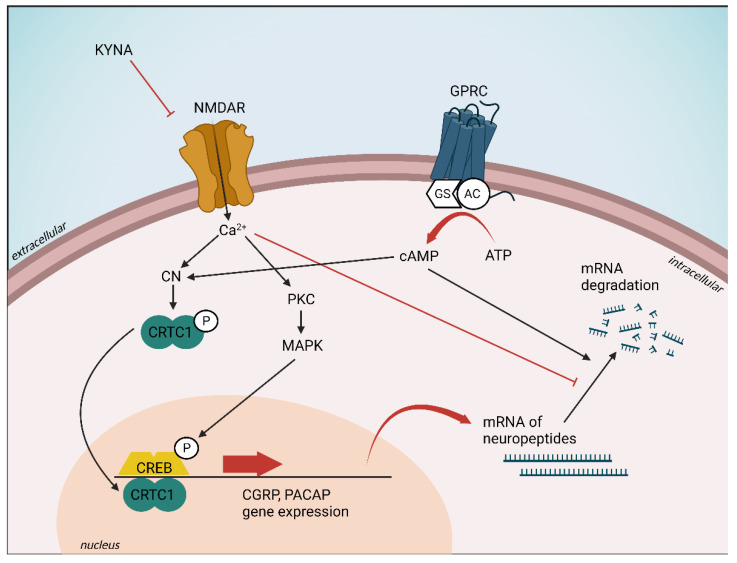
Proposed regulation of CGRP and PACAP gene expression. AC: adenylate cylase, ATP: adenosine monophosphate, CaM: calmodulin, cAMP: cyclic adenosine monophosphate, CGRP: calcitonin gene-related peptide CN: calcineurin, CREB: cAMP response element-binding protein, CRTC1: CN/Cre-binding protein, GPCR: G-protein-coupled receptor, Gs: stimulatory G protein, KYNA: kynurenic acid, MAPK: mitogen-activated protein kinase, NMDAR: NMDA receptor, PACAP: pituitary adenylate cyclase activating polypeptide PKC: protein kinase C.

The main mediator of CGRP and PACAP gene expression is intracellular calcium homeostasis. In addition to the action of voltage-dependent calcium channels, the main inducer of the gene expression of these peptides is calcium influx through the NMDA receptors. KYNA and its analogs can block the NMDA receptors, thereby moderating the amount of calcium coming into the cell, which may result in decreased CGRP and PACAP gene expression. Since KYNA and its analogs can decrease migraine-related neuropeptides expression, targeting CGRP and PACAP with KYNA may have a therapeutic role in the future (Figure 6). In a previous experiment by Körtési et al., the expression levels of PACAP were significantly different between the uncompetitive antagonist of the NMDA glutamate receptor MK-801 and SZR-72 treatment groups, raising the possibility of the involvement of KYNA targets other than NMDA. In addition to NMDA receptor, KYNA has an effect on the AMPA, kainate, aryl hydrocarbon, GPR35, and opiate receptors. Regarding SZR-72, investigations are in process, but the exact targets and mechanisms of this analog have not yet been identified [127].

## 6. Clinical Studies

Several lines of evidence suggest that imbalance of the kynurenine pathway plays roles in several diseases [105]. Several preclinical studies reflect a link between the kynurenine pathway and migraine. Indeed, numerous studies have demonstrated that the NMDA receptor inhibitor KYNA and its analogs have anti-nociceptive effects at the levels of both first- and second-order sensory neurons [19]. KYNA and one of its derivatives both decreased the levels of several inflammatory mediators in the animal model of CFA-induced TS activation [145]. The effects of two KYNA analogs were tested in the orofacial formalin model, revealing that both were able to inhibit the formalin-induced behavioral and morphological changes, as well as increase the concentration of KYNA in the rat brainstem [150]. Notably, systemic administration of NTG decreased the expression of KAT II in the TS of rats, an enzyme catalyzing the transformation of L-KYN to KYNA [151]. In line with this, in another model of TS activation, decreased KAT immunoreactivity was observed in mast cells, Schwann cells, and dural macrophages [113]. In addition to preclinical studies, clinical results have provided evidence for the connection between the kynurenine system and various headache disorders, including migraine or cluster headache. Indeed, in patients suffering from primary headache disorders, alterations of the kynurenine pathway were observed, which, among others, manifested in the reduction in KYNA concentration in the serum [25,26]. A clinical study also proved that plasma concentrations of most tryptophan metabolites were remarkably decreased in the interictal period of migraineurs compared to healthy control subjects, especially in the migraine without aura subgroup (tryptophan, L-KYN, KYNA, ANA, PIC, 5-HIAA, and melatonin). In patients suffering from migraine without aura, several metabolites showed a tendency to elevate during the ictal phase, but this was significant only in the cases of ANA, 5-HIAA, and melatonin [152]. A clinical phase I investigation proved that intravenous administration of L-KYN is safe and well tolerated. The lack of change in kynurenine metabolites in plasma reflects a relatively slow metabolism of L-KYN and no or little feed-back effect of this metabolite on its synthesis [153].

## 7. Conclusions

The aim of the present work was to draw some attention to the role of different tryptophan catabolites; furthermore, we were able to gain insight into the role of various neuropeptides in the pathomechanism of migraine. The serotonin and kynurenine pathways are closely connected, and alterations in one arm of the pathway may influence the other. Tryptophan metabolites play an important role in primary headaches, and they can be potential therapeutic targets in the treatment of the migraine and other primary headaches.

Neuropeptides, CGRP and PACAP in particular, are implicated in trigeminal activation. The expression of CGRP and PACAP and its receptors, and their main effects and mechanisms in the nociceptive pathways suggest that these neuropeptides have a special role in migraine. Identification of their molecular mechanisms might open up future perspectives for the development of novel analgesic drugs.

## Abbreviations

3-HANA	3-hydroxyanthranilic acid
3-HAO	3-hydroxyanthranilate oxidase
3-HK	3-hydroxy-L-kynurenine
5-HIAA	5-hydroxyindoleacetic acid
5-HT	serotonin
5-HTP	5-hydroxytryptophan
5-HTT	serotonin transporter
AA	anthranilic acid
AADC	L-aromatic amino acid decarboxylase
AMPA	α-amino-3-hydroxy-5-methyl-4-isoxazole propionic acid
ATP	adenosine triphosphate
BBB	blood–brain barrier
BDNF	brain-derived neurotrophic factor
CFA	complete Freund’s adjuvant
CGRP	calcitonin gene-related peptide
CNS	central nervous system
CSD	cortical spreading depression
DRG	dorsal root ganglion
ERK	extracellular signal-regulated kinase
FHM	familial hemiplegic migraine
GABA	gamma-aminobutyric acid
GFAP	glial fibrillary acidic protein
GPR35	G-protein-coupled receptor 35
Iba1	ionized calcium-binding adapter molecule 1
IDO	indoleamine-2,3-dioxygenase
IL-1 β	interleukin-1
KAT	kynurenine aminotransferase
KMO	kynurenine-3-monooxygenase
KP	kynurenine pathway
KYNA	kynurenic acid
KYNU	kynureninase
L-KYN	L-kynurenine
MAO	monoamine oxidase
MAPK	mitogen-activated protein kinases
MCA	middle cerebral artery
mCPP	m-chlorophenylpiperazine
MMA	middle meningeal artery
MT1/2	melatonin receptors 1/2
NAD	nicotinamide adenine dinucleotide
NADP	nicotinamide adenine dinucleotide phosphate
NMDA	N-methyl-D-aspartate
NO	nitric oxide
NOS	nitric oxide synthases
NTG	nitroglycerin
PACAP	pituitary adenylate cyclase-activating polypeptide
PIC	picolinic acid
PPE	plasma protein extravasation
QUIN	quinolinic acid
SP	substance P
TDO	tryptophan-2,3-dioxygenase
TG	trigeminal ganglia
TMJ	temporomandibular joint
TNC	trigeminal nucleus caudalis
TPH	L-tryptophan-hydroxylase
TRP	tryptophan
TRPV1	transient receptor potential vanilloid 1
TS	trigeminal system
TSH	thyroid-stimulating hormone
VIP	vasoactive intestinal peptide
VMAT2	vesicular monoamine transporter isoform 2
XA	xanthurenic acid
